# Effect of the COVID-19 pandemic on child development: comparing pre- and post-pandemic developmental process levels

**DOI:** 10.3389/fpsyg.2026.1703384

**Published:** 2026-04-10

**Authors:** Takashi Otani, Masaharu Kato, Hisami Haraguchi, Hideyo Goma

**Affiliations:** 1 Department of Psychology, Faculty of Health Science, Kyoto Koka Women’s University, Kyoto, Japan; 2 Japan Developmental Cohort Research and Application Association, Kyoto, Japan; 3 Department of Childcare, Kindai University Kyushu Junior College, Fukuoka, Japan; 4 Department of Nursing, Faculty of Nursing, Himeji University, Hyogo, Japan

**Keywords:** child development, COVID-19 pandemic, follow-up study, language development, post-pandemic

## Abstract

Concerns about the potential impact of the COVID-19 pandemic on children’s development have prompted numerous developmental studies during the pandemic period. However, few studies have examined children’s developmental trajectories beyond the lifting of pandemic-related social restrictions. This study examined the developmental progress of children aged 36–42 months, who experienced the COVID-19 pandemic during their early developmental period, as a follow-up to its previous study, which evaluated the developmental progress of the same children when they were 18–24 months during the pandemic. When comparing pre-, during-, and post-COVID data, the findings confirmed that the effects on language development previously observed in these children at 18–24 months had disappeared by the time they reached the 36–42-month investigation. However, the findings at 36–42 months also revealed a decline in the developmental quotient for motor development, marking the first time such an effect has been observed in a longitudinal study. Further verification is needed to determine whether this decline during the early stages of development was due to the pandemic or other factors.

## 1 Introduction

The COVID-19 pandemic has led to substantial lifestyle changes, such as school closures and official restrictions, which may have affected children’s development. Although studies on the effects of such changes have yielded mixed results depending on the research methodology, geographic region, and participants’ age, they consistently suggest a negative impact on child development (Araújo et al., 2021; Hessami et al., 2022; Betthauser et al., 2023), particularly in the domain of language development (Huang et al., 2021; Frota et al., 2022; Nevo, 2024; Otani et al., 2024; Zuniga-Montanez et al., 2025). With the advancement of vaccines and therapeutic treatments, various social restrictions were gradually eased and eventually lifted, allowing daily life to return to its pre-pandemic state. Nevertheless, COVID-19-related school closures and voluntary absences may have had lingering effects. For example, Makhluf et al. (2024) reported that long COVID continues to affect more than 65 million individuals globally, with symptoms ranging from chronic fatigue syndrome and brain fog to autoimmune dysfunction and cardiovascular complications. Basaca et al. (2025) reported the effects of long COVID on children’s psychological symptoms and cognitive development through a systematic review of over 50 studies. Goulas and Pula (2024) emphasized that school attendance had not returned to pre-pandemic levels in many countries. However, most studies focused on the effects of long COVID; few studies examined the long-term effects of COVID-19 restrictions on child development post-pandemic.

A previous study had conducted a developmental study during the COVID-19 pandemic to clarify the effects of the pandemic in the Japanese context, and assess developmental trajectories using individualized developmental scales from 10–11 to 18–24 months of age (Otani et al., 2024). It found a decline in the developmental quotient (DQ) in the Language–Social (L–S) area, although whether this effect persists beyond 24 months of age remains unverified. To build on Otani et al.’s (2024) study, and address gaps in post-pandemic research regarding child development, this study aimed to investigate whether the decline in DQ observed in the L–S area at 18–24 months during the COVID-19 pandemic (Otani et al., 2024) would persist at 36–42 months of age post-pandemic. The COVID-19 pandemic has been reported to primarily affect children’s language development, with these impacts potentially becoming long-term (Benner and Mistry, 2020; Anunciacao et al., 2025). Therefore, this study also aimed to investigate the developmental trajectories of children who exhibited language-related effects at 18–24 months to address this concern. By analyzing longitudinal data across multiple developmental stages, and comparing them with standardized pre-pandemic norms, it sought to clarify the extent and duration of the pandemic-related developmental impacts.

## 2 Materials and methods

### 2.1 Study design

**FIGURE 1:**
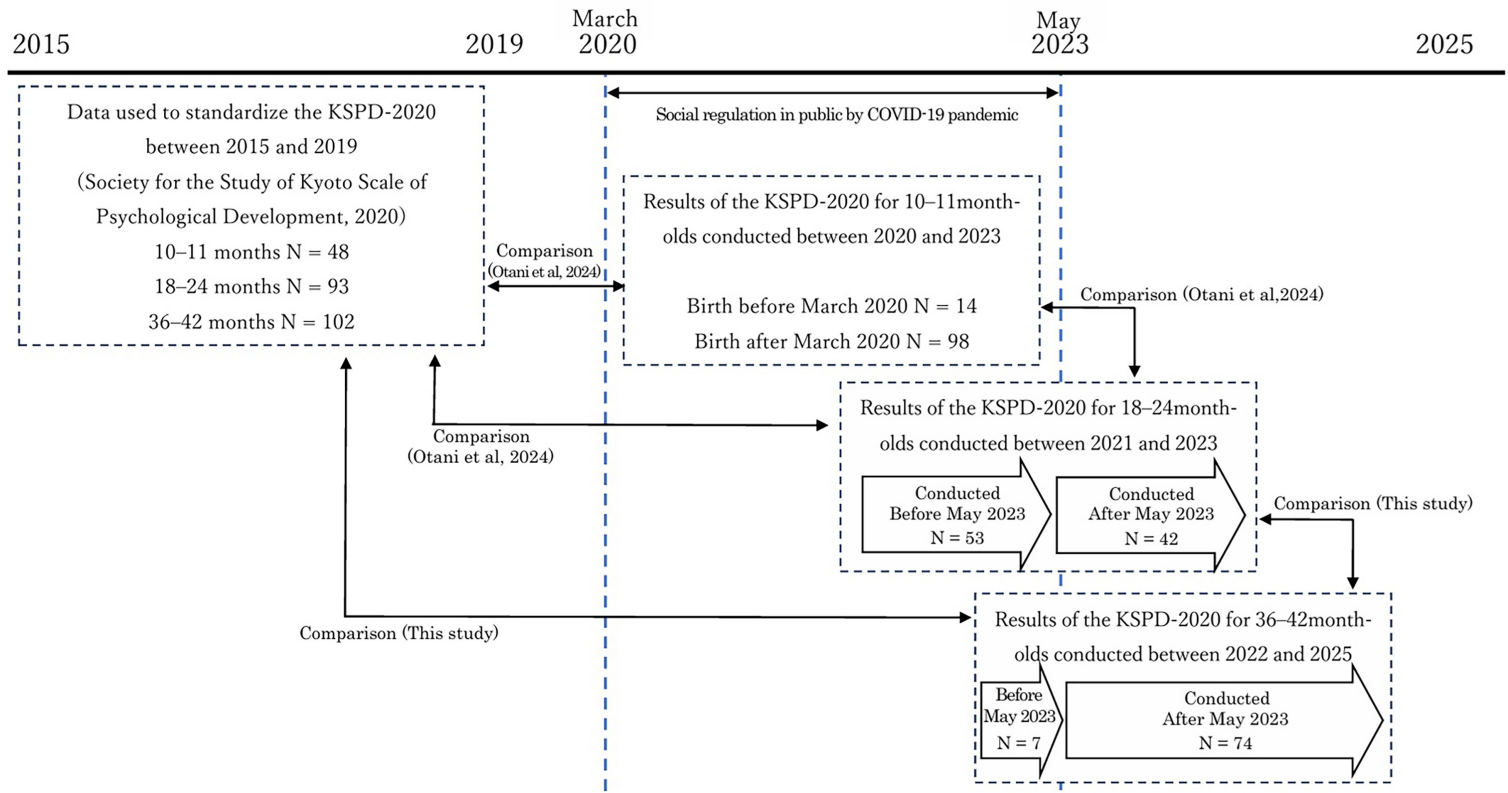
Study design.

During the pandemic (2020–2023), Otani et al. (2024) evaluated the developmental state of children at 10–11 months, using an individualized developmental scale. They later conducted a follow-up evaluation from June 2021–December 2023, after the children had reached 18–24 months of age. This study continues this work by evaluating the developmental state of the same children after they reached 36–42 months of age during the post-pandemic period in Japan, which was after the legal classification of COVID-19 in Japan was downgraded to the same level as that of seasonal influenza in May 2023. Developmental processes post-COVID were compared with those during-COVID levels (Otani et al., 2024), as well as pre-pandemic-level estimates (2015–2019) for developmental processes according to the standardized data from the Kyoto Scale of Psychological Development (KSPD)-2020 (Society for the Study of the Kyoto Scale of Psychological Development, 2020). The study design is illustrated in Figure 1.

This study has two primary objectives. First, to determine whether the decline in language and social development indices observed at the 18–24-month assessment reported by Otani et al. (2024) persisted at the 36–42-month assessment. Second, to clarify the impact of the COVID-19 pandemic on child development by comparing the current data with pre-pandemic records. For this purpose, it examined how developmental indices across developmental areas changed from 18–24 months to 36–42 months among children who participated in both assessments, and for whom complete data were available. A mixed-model analysis was conducted, with assessment ages and assessment areas as independent variables, and the DQ for each development area as the dependent variable. To evaluate these variables’ main effects and interactions mixed-effects models with increasing complexity, were constructed. In all models, age at investigation, development areas, and their interactions, were treated as fixed effects, whereas individual differences were treated as random effects. Parental age, employment status, and household income were not included in the analyses for two reasons. First, in Japan, such information is considered highly private, and requesting it could have discouraged participation. Second, the pre-pandemic standardization data from the Kyoto Scale of Psychological Development-2020 (KSPD-2020), which served as this study’s comparison baseline, also did not collect these variables, making it impossible to control for them in pre-pandemic comparisons. The implications of this limitation are discussed in the “5 Conclusions and limitations” section.

For comparisons with pre-pandemic data, this study used the standardized data from KSPD-2020 along with the data collected in this study. However, the standardized dataset provided only the mean and standard deviation (SD) of DQ scores for each age group, and raw data were not available. Therefore, for assessments of the DQ scores in each of the developmental areas at both 18–24 and 36–42 months, independent *t*-tests using investigation timing as the independent variable, and the DQ for each developmental area as the dependent variable was conducted. This analysis examined whether mean DQ scores differed between the pre-pandemic data and this study. The Bonferroni method was applied to correct for multiple comparisons. Following Otani et al.’s (2024) approach, this study continued to exclude the full-scale DQ from between-group comparisons because it was not independent, and included the results of the three developmental areas in varying proportions.

### 2.2 Participants

This study included 81 participants (41 boys and 40 girls) from Otani et al.’s (2024) study, which involved participants aged between 36 and 42 months. As with the investigation at 18–24 months, the assessments at 36–42 months were conducted in the research room of the Center for Baby Science, Doshisha University. The research period was between November 2022 and February 2025. All participants were born between October 2019 and May 2022, indicating that their early developmental period coincided with the COVID-19 pandemic. The average ages at the 18–24 and 36–42-month assessment periods were 588.7 days (SD = 39.9) and 1,131.5 days (SD = 34.7), respectively. All children were born at a gestational age of over 37 weeks, and had birth weights exceeding 2,500 grams. No significant medical complications were observed. The average maternal and paternal ages at childbirth were 32.9 years (SD = 3.9), and 34.0 years (SD = 4.7), respectively. These characteristics suggest that the developmental data collected at both time points reflect children with generally typical birth conditions.

Of the 95 participants assessed at 18–24 months, 14 were unable to participate in the 36–42-month assessment owing to difficulties in continuing participation caused by relocation or loss of contact. Notably, 10 participants were unable to continue because the Doshisha University Center for Baby Science, where the investigations were conducted, closed at the end of March 2025.

### 2.3 Measures

Two instruments were used for developmental assessment: KSPD-2020—an individualized, examiner-administered scale—and the Kinder Infant Developmental Scale (KIDS) Type C (a parent-report questionnaire for children aged 36–72 months). Regarding KIDS, when no items were checked in any domain of Type C, Type B (for children aged 12–36 months) was used to account for lower developmental age. However, Type B was applied to only one domain for 2 of the 81 participants, indicating extremely limited use. Consequently, the developmental ages measured by KIDS exceeded the chronological ages for all participants across all tests. This suggests that the combined use of Types C and B failed to function appropriately, meaning the data from this survey could not adequately assess developmental status for children under 3 years of age. Therefore, the results of KIDS were excluded from the analysis in this study. Consequently, only the KSPD-2020 data were used to analyze developmental trajectories from the pre- to post-pandemic periods.

#### 2.3.1 KSPD-2020

The KSPD-2020—a standardized, individualized developmental assessment tool widely used in Japan—is applicable from infancy to adulthood, although it is primarily used to evaluate child development. The scale comprising 339 test items and some sub-items is administered in a one-on-one format. The testing duration typically ranges from 30 to 40 min for infants and toddlers, and over an hour for school-aged children and adults. The KSPD-2020 was standardized using data from 3,243 individuals collected between 2015 and 2019. Its reliability and validity were confirmed through retest methods and comparison with the Wechsler Adult Intelligence Scale, Third Edition, yielding correlation coefficients of *r* = 0.69 and *r* = 0.75, respectively (Society for the Study of the Kyoto Scale of Psychological Development, 2020). Although the sampling included all regions of Japan, the representation from western areas, including Kyoto, was relatively higher. Furthermore, although adjustments were made to balance the ratio of urban and rural areas, data on parental age and income were not collected, and the sample was randomly selected. The scale provides DQs for three development areas—Postural–Motor (P–M), Cognitive–Adaptive (C–A), and L–S—along with an overall full-scale DQ. The DQ of the KSPD-2020 is calculated as the ratio of developmental age to chronological age.

### 2.4 Data analysis

This study used IBM SPSS 26.0 to conduct *t*-tests and analysis of variance (ANOVA) to determine whether there were differences in the mean DQ. For the mixed-effects models, it used R version 4.4.1 with the nlme package version 3.1-165.

### 2.5 Ethical considerations

As this study is a follow-up to Otani et al. (2024), it applied the same ethical procedures, which included obtaining informed consent from all caregivers, and securing approval from the Research Ethics Review Committees for Human Participants at Doshisha University (Approval No. 2-7), Kyoto Koka Women’s University (Approval No. 113), and Nara University of Education (Approval No. 18003). Prior to participation, the caregivers were provided with both verbal and written explanations regarding the study’s purpose, procedures, voluntary nature of participation, and handling and anonymization of the data. Informed consent was obtained from all participants. The collected data were anonymized in a linkable manner, allowing identification only when necessary, such as in cases involving reward payments or data withdrawal requests.

## 3 Results

### 3.1 Internal consistency and test–retest stability

Cronbach’s alpha coefficients were calculated across developmental areas to verify the internal consistency at 18–24 and 36–42 months. DQ coefficients at 18–24 months were 0.69 for P–M, 0.86 for C–A, and 0.66 for L–S. At 36–42 months, coefficients were 0.49 for P–M, 0.81 for C–A, and 0.88 for L–S. The KSPD’s 4-year, 4-month evaluative ceiling for motor development may have affected the lower P–M alpha coefficient observed at the 36–42-month assessment.

Furthermore, to verify test–retest stability, correlation coefficients were calculated for each DQ domain between the 18–24 and 36–42-month assessments. The resulting values—*r* = 0.35 for P–M, *r* = 0.33 for C–A, and *r* = 0.38 for L–S—were lower than those reported during standardization. This discrepancy may be attributed to the participants’ young age (under 2 years old at the first assessment) combined with the long intervals between the two assessment points.

### 3.2 Developmental processes at 18–24 months versus at 36–42 months

The KSPD results for 18–24 and 36–42 months are shown in Table 1. At the 18–24-month follow-up, mean DQs were 99.7 for P–M (SD = 17.2), 98.0 for C–A (SD = 14.5), and 93.0 for L–S (SD = 14.0), whereas by 36–42 months, these scores shifted to 94.8 for P–M (SD = 17.6), 98.9 for C–A (SD = 12.5), and 97.5 for L–S (SD = 11.6).

**TABLE 1:** Results of the Kyoto Scale of Psychological Development-2020 at 18–24 and 36–42 months.

Developmental areas	*N*	18–24 months		36–42 months		Multiple comparisons
		Average age (days)	DQ (SD)	Average age (days)	DQ (SD)	
P–M	81	588.7 (39.9)	99.7 (17.2)	1,131.5 (34.7)	94.8 (17.6)	*t* (80) = 2.19, *p* = 0.032, *d* = 0.282, 95% CI [−0.28, 0.59]
C–A			98.0 (14.5)		98.9 (12.5)	*t* (80) = −0.49, *p* = 0.627, *d* = −0.070, 95% CI [−0.37, 0.24]
L–S			93.0 (14.1)		97.5 (11.6)	*t* (80) = −2.47, *p* = 0.015, *d* = −0.349, 95% CI [−0.66, −0.04]

Multiple comparisons were adjusted using the Bonferroni method (*m* = 3).

To investigate the effects of age at investigation and development areas on DQ, this study primarily focused on two models based on its sample size (*n* = 81). Model 1, which accounted for individual differences in the intercept, and Model 2, which accounted for individual differences in the intercept and age at investigation. It also explored more complex random effect structures. One where individual differences influenced both the intercept and development areas, and another where they affected the intercept, age at investigation, and development areas. However, as anticipated, these two models encountered singularity issues due to the limited sample size relative to their complexity. A model comparison using Akaike Information Criterion showed that Model 2 had a higher goodness of fit (3,960.1) than Model 1 (3,960.2). Subsequently, a type III ANOVA using Satterthwaite’s method was performed on Model 2 to assess the significance of each fixed effects term within the model. The results of the ANOVA showed that the interaction effect of the age at investigation and the development area was significant [*F*(2, 320) = 5.76, *p* = 0.0035, η_p_^2^ = 0.03]. A simple main effect test for the interaction using the Bonferroni method (*m* = 3) showed that the mean DQ of L–S in the investigation at 36–42 months was significantly higher than that at 18–24 months {*t* (80) = 2.19, *p* = 0.032, d = 0.281, 95% CI [−0.28, 0.59]}, and the mean DQ of P–M in the investigation at 36–42 months was significantly lower than that at 18–24 months {*t* (80) = −2.47, *p* = 0.015, d = 0.349, 95% CI[−0.66, −0.04]}.

### 3.3 Comparison with pre-pandemic data

In the pre-pandemic data (Society for the Study of the Kyoto Scale of Psychological Development, 2020), the mean DQs of the developmental areas at 18–24 months of age were 102.1 for P–M (SD = 9.7), 102.1 for C–A (SD = 18.4), and 98.1 for L–S (SD = 12.4), and the mean age of the participants (48 boys and 45 girls) was 641.7 days. Regarding DQ data at 36–42 months of age, the means were 99.0 for P–M (SD = 16.1), 99.7 for C–A (SD = 13.1), and 99.8 for L–S (SD = 12.1), and the mean age of the participants (60 boys and 47 girls) in the pre-pandemic data at 36–42 months was 1,181.2 days. A *t*-test using the Bonferroni method (*m* = 6) was used to confirm whether the mean DQ of each developmental area at 18–24 and 36–42 months differed significantly between 2015 and 2019 data and this study’s data, for which no significant differences were found (Table 2).

**TABLE 2:** Kyoto Scale of Psychological Development-2020 result comparisons for 18–24-month-olds pre- (2015–2019) and during-pandemic (2021–2025).

Chronological age at assessment	Developmental areas	Pre–pandemic data		This study		*t*-test
		*N*	Average DQ (SD)	*N*	Average DQ (SD)	
18–24 months	P–M	93	102.1 (9.7)	81	99.7 (17.2)	*t* (172) = 1.15, *p* = 1.00, *d* = 0.175, 95% CI [−0.12, 0.47]
	C–A		102.1 (18.4)		98.0 (14.5)	*t* (172) = 1.62, *p* = 0.648, *d* = 0.246, 95% CI [−0.05, 0.55]
	L–S		98.1 (12.4)		93.0 (14.1)	*t* (172) = 2.57, *p* = 0.065, *d* = 0.386, 95% CI [0.09, 0.69]
36–42 months	P–M	107	99.0 (16.1)	81	94.8 (17.6)	*t* (186) = 1.70, *p* = 0.543, *d* = 0.250, 95% CI [−0.04, 0.54]
	C–A		99.7 (13.1)		98.9 (12.5)	*t* (186) = 0.42, *p* = 1.00, *d* = 0.062, 95% CI [−0.23, 0.35]
	L–S		99.8 (12.1)		97.5 (11.6)	*t* (186) = 1.31, *p* = 1.00, *d* = 0.194, 95% CI [−0.10, 0.48]

Multiple comparisons were adjusted using the Bonferroni method (*m* = 6).

## 4 Discussion

### 4.1 Comparison of results from the 18–24-month and 36–42-month assessments

Comparing DQ scores across developmental areas between the 18–24-month and 36–42-month assessments revealed that the L–S DQ scores were significantly lower during the 18–24-month assessment, but stabilized by the 36–42-month follow-up, where no decline in L–S DQ was observed. In contrast, a decline in P–M DQ scores were evident at the 36–42-month assessment. The subsequent paragraphs, first examine the results related to language development, followed by those concerning motor development.

The delay in children’s language development observed during the COVID-19 pandemic is consistent with earlier research. Specifically, the language development delay observed at 18 months of age—a critical stage for language acquisition—corresponds with the findings of Matsuo et al. (2025) in Japan. This study did not collect information on children’s living conditions (e.g., preschool attendance) or parental socioeconomic factors, making it difficult to draw definitive conclusions. For example, Matsuo et al. (2025) and Feijoo et al. (2023) indicated that reduced opportunities for social interaction contributed to delays in language development, with the impact being especially marked among children cared for at home (i.e., those who did not attend nursery or preschool). In Japan, the utilization rate of nursery and preschool services was 45% for one-year-olds, whereas it reached 95% for three-year-olds (Japan Children and Families Agency Establishment Preparation Office, 2023). As the participants’ enrollment status remained unconfirmed in this study, definitive conclusions could not be drawn. Moreover, the possibility that changes in nursery or preschool attendance between the 18–24-month and 36–42-month assessments may have influenced the results warrants consideration.

Furthermore, the 36–42-month assessment confirmed a decline in the P–M DQ scores. Rigó and Weyers (2024) noted that social factors, including the home environment, were associated with the impact of the COVID-19 pandemic on motor development, reporting that children raised in more affluent households actually exhibited a more pronounced decline. Abe et al. (2022) noted a marked decline in children aged 5 years and above, despite finding no significant impact on motor development in 3–4-year-olds. This suggests that the effects of pandemic-related restrictions may not be immediately observable during infancy, when motor activity is primarily bilateral and less complex. Dudek and Zalazny (2007) found a relationship between the duration of crawling in infancy and subsequent development of spatial cognition and visual search, suggesting that the nature of motor activity during infancy may influence later developmental trajectories. However, Johnson et al. (2024) reported that preschool children’s fine and gross motor skills remained unchanged during the COVID-19 pandemic. Therefore, the findings of these studies are inconsistent. This study too, cannot provide further discussion on that factor, as it did not obtain data on the children’s home environment. Hence, sustained investigation into children’s motor development in the post-pandemic era is warranted, specifically by incorporating data on their home and child-care environments.

### 4.2 A comparison with pre-pandemic data

When compared with pre-pandemic data, no significant differences were observed in any developmental areas at either the 18–24 or 36–42-month assessments. The reason for the cross-sectional comparison with pre-pandemic data yielding different results from the analysis in the previous section might have been due to differences in sampling across the studies. This study’s participants were limited to parents and their children, who were able to visit the Doshisha University Center for Baby Science, the location of this study. In comparison, the pre-pandemic data from the KSPD-2020 were collected nationwide in Japan. In addition, differences in research design—a longitudinal study versus a cross-sectional study—may have influenced the results. Longitudinal studies that follow the same individuals over time are more sensitive to intra-individual developmental changes and cumulative environmental influences. In contrast, assessing different individuals may mask subtle developmental shifts. As Getchell et al. (2020) emphasize, longitudinal designs are particularly well-suited for detecting developmental trajectories and delayed effects, especially in motor development research. In previous studies examining the impact of the pandemic, questionnaires have frequently been used to assess children’s development. Conversely, although this study involved a small, regionally limited sample, its strength lies in its being a longitudinal investigation, that utilized developmental assessments based on individualized tests administered by specialists.

The impact of the pandemic has been a major concern for developmental support professionals, educators, and parents, with significant attention focused on the extent of its effects and the post-pandemic developmental trajectory. This study examined the developmental trajectory of language-related impacts observed in children aged 18–24 months during the COVID-19 pandemic and confirmed that these effects were no longer detectable by age 3. This finding has important implications for understanding recent trends in child development. First, regarding the long-term effects of the COVID-19 pandemic, the results of this study can be compared with findings from other regions and other evaluation scales. Furthermore, it suggests the importance of comprehensively examining children’s current developmental profiles and the factors influencing them, without overemphasizing the pandemic’s impact, while also considering the effects of changes in children’s living environments that predate the pandemic, such as increased screen time. This study’s results indicate only that no effects were observed on language abilities assessed using a developmental scale. Concerns persist that the substantial reduction in opportunities for sociocultural activities during the early childhood years may have long-term effects on children’s psychological and social development (Anunciacao et al., 2025), necessitating continuous monitoring and follow-up.

## 5 Conclusion and limitations

This study’s results revealed longitudinal data on the development of children born between October 2019 and May 2022, who experienced the COVID-19 pandemic during their early developmental stages. One hypothesis is that the decline in DQ in L–S skills observed at 18–24 months was no longer evident at 36–42 months, suggesting a potential recovery following the lifting of social restrictions. In contrast, a decline in DQ in P–M skills were observed at 36–42 months. This finding aligns with recent studies indicating that motor delays may emerge later, specifically, when more complex coordination and bilateral integration are expected as part of typical development.

Given that longitudinal studies that assess the same children through face–to–face developmental evaluations during and after the pandemic are extremely rare, this study’s findings are invaluable for verifying the recently observed changes in child development. However, this study did have several limitations. First, no data existed on the participants’ history of COVID-19 infections because at the time of this study, such information was highly sensitive, given that, it could lead to prejudice or exclusion from the community. Therefore, the impact of long COVID on child development was not considered when interpreting its results. Second, the sample size was small because it was extremely difficult to secure participants willing to participate in face–to–face surveys during the COVID-19 pandemic. Although 112 participants were recruited for the initial investigation when they were 10–11 months old, many later dropped out of subsequent studies because their families relocated. Additionally, the study sample was largely drawn from the Kyoto Prefecture and surrounding areas, and participation was limited to families, who were motivated and willing to participate in face–to–face research despite the pandemic, which may limit the generalizability of the study’s findings to the broader Japanese population. Furthermore, this study did not collect information on social confounding factors, such as the age of participants’ caregivers, their social class, or their employment status; hence, these variables were not included in the mixed-effects model analysis. In Japan, this type of information is considered highly private, and collecting it could have discouraged individuals from participating in the study. In fact, pre-pandemic research using random sampling, did not collect this data either. The absence of these variables limits this study’s ability to determine whether the observed developmental patterns were influenced by sociodemographic factors that may have changed during the pandemic period (e.g., parental employment disruptions, changes in household income). Future research with more comprehensive demographic data would help clarify whether the observed associations are independent of these potential confounders.

## Data Availability

The datasets presented in this article are not readily available because the dataset cannot be provided to third parties because participants’ consent has not been obtained for any purpose other than the original study. However, if a situation arises in which objective confirmation of the research content is required, we are prepared to disclose the data for appropriate confirmation. Requests to access the datasets should be directed to Takashi Otani, t-otani@mail.koka.ac.jp.
